# Characterization of the Phytochelatin Synthase of *Schistosoma mansoni*

**DOI:** 10.1371/journal.pntd.0001168

**Published:** 2011-05-24

**Authors:** Debalina Ray, David L. Williams

**Affiliations:** 1 Department of Biological Sciences, Illinois State University, Normal, Illinois, United States of America; 2 Department of Immunology/Microbiology, Rush University Medical Center, Chicago, Illinois, United States of America; McGill University, Canada

## Abstract

Treatment for schistosomiasis, which is responsible for more than 280,000 deaths annually, depends exclusively on the use of praziquantel. Millions of people are treated annually with praziquantel and drug resistant parasites are likely to evolve. In order to identify novel drug targets the *Schistosoma mansoni* sequence databases were queried for proteins involved in glutathione metabolism. One potential target identified was phytochelatin synthase (PCS). Phytochelatins are oligopeptides synthesized enzymatically from glutathione by PCS that sequester toxic heavy metals in many organisms. However, humans do not have a PCS gene and do not synthesize phytochelatins. In this study we have characterized the PCS of *S*. *mansoni* (SmPCS). The conserved catalytic triad of cysteine-histidine-aspartate found in PCS proteins and cysteine proteases is also found in SmPCS, as are several cysteine residues thought to be involved in heavy metal binding and enzyme activation. The SmPCS open reading frame is considerably extended at both the N- and C-termini compared to PCS from other organisms. Multiple PCS transcripts are produced from the single encoded gene by alternative splicing, resulting in both mitochondrial and cytoplasmic protein variants. Expression of SmPCS in yeast increased cadmium tolerance from less than 50 µM to more than 1,000 µM. We confirmed the function of SmPCS by identifying PCs in yeast cell extracts using HPLC-mass spectrometry. SmPCS was found to be expressed in all mammalian stages of worm development investigated. Increases in SmPCS expression were seen in *ex vivo* worms cultured in the presence of iron, copper, cadmium, or zinc. Collectively, these results indicate that SmPCS plays an important role in schistosome response to heavy metals and that PCS is a potential drug target for schistosomiasis treatment. This is the first characterization of a PCS from a parasitic organism.

## Author Summary

Schistosomiasis is a chronic, debilitating disease that affects hundreds of millions of people. The treatment of schistosomiasis relies solely on monotherapy with praziquantel and there is concern that drug-resistant parasites will evolve. Therefore, it is imperative to identify new drugs for schistosomiasis treatment. In this study our goal was to characterize a unique gene of *Schistosoma mansoni* that may be a candidate for drug targeting to control schistosomiasis. This gene, phytochelatin synthase (PCS), is a single copy gene in *S*. *mansoni* but is absent from humans. Our results confirm that schistosome PCS produces phytochelatins that are capable of scavenging and detoxifying heavy metals. The expression of the PCS gene in *ex vivo* adult schistosome worms was increased by exposure to a number of heavy metals. These results indicate that *S*. *mansoni* PCS regulates the availability of metal ions that the worm may be exposed to, either as co-factors in metalloenzymes or as excess metals encountered in the blood stream of their mammalian host. Collectively, these results have important implications for drug development for the control of schistosomiasis. Since other helminth parasites have PCS, drug development targeting this enzyme may have wide applications in the control of multiple neglected diseases.

## Introduction

Schistosomiasis is a chronic disease caused by trematode flatworms of the genus *Schistosoma*. This neglected, poverty-related disease is found in more than 70 countries. It is estimated that more than 200 million people are afflicted with schistosomiasis, with 779 million at risk of infection, resulting in 280,000 deaths annually [1]. Currently only one drug, praziquantel, is used against schistosomiasis. The low cost of the drug and its efficacy against adult worms of all schistosome species that infect humans has led to its widespread use; currently tens of millions receive annual treatments of PZQ [2]. However, because of high re-infection rates drugs must be administered on an annual or semi-annual basis. It is speculated that the exclusive use of a single drug should hasten the evolution of drug resistant parasites [2]. In the laboratory, *S*. *mansoni* subjected to drug pressure can develop resistance to praziquantel over the course of relatively few passages [3]. There are clinical reports of praziquantel failures in *S. mansoni*- and *S. haematobium-*endemic areas of Senegal and Egypt and Kenya [4], [5], [6]. The availability of alternatives to praziquantel is extremely limited; they are more expensive, have unacceptable side effects and/or are effective on only one schistosome species [7]. Therefore, there is an urgent need to identify new targets and drugs for schistosomiasis treatment.

A pathogen protein can be considered a good drug target if it is essential for pathogen survival, unique to the pathogen and drugable. We hypothesized that one such potential target in schistosomes is phytochelatin synthase (PCS). Phytochelatins (PCs) are a family of peptides that chelate heavy metals [8], [9]. They are synthesized non-translationally by PCS from glutathione (GSH), a tripeptide, (γ-glutamic acid-cysteine-glycine or γ-Glu-Cys-Gly or γ-ECG) [10], [11], that is itself synthesized by two enzymes γ-glutamyl-cysteine ligase and GSH synthase [12]. GSH is the most abundant low molecular weight thiol in most cells, provides protection against oxidative damage and plays important roles in cell proliferation, redox regulation of gene expression, xenobiotic metabolism, and several other metabolic functions [13].

Phytochelatins have the general formula (γ-EC)_n_-G, where n = 2–11, and are formed by the transfer of the γ-EC dipeptide from one GSH to a second GSH with the release of glycine [10], [11]:



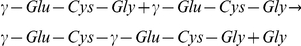



PCS proteins are γ-EC dipeptidyl transpeptidases (EC 2.3.2.15) [11] and belong to the papain superfamily of cysteine proteases with conservation of the 3D geometry of the catalytic Cys-His-Asp triad [14], [15]. Recently PCS have been identified in a number of additional organisms. *Caenorhabditis elegans* has a single copy PCS gene that confers cadmium resistance when heterologously expressed in yeast [16]. Silencing by RNA interference of *C*. *elegans* PCS lead to a cadmium-hypersensitive phenotype [16]. PCS genes or transcripts have been identified in the parasitic nematodes *Brugia malayi* and *Parascaris univalens* [17] and in other metazoan organisms, including lower chordates [18] and in several prokaryotic genomes [18], [19], [20], [21]. In contrast, there are no PCS genes encoded in the genomes of mammals, which instead use GSH and metallothioneins, low molecular weight, Cys-rich proteins, to regulate the availability of heavy metals [22].

A number of heavy metals (e.g., Fe, Zn, Cu, Mn) are essential micronutrients for most organisms, and are involved in the catalytic activity or structural stability of numerous enzymes. However, an excess of these heavy metals is often toxic and their cellular levels must be tightly controlled. For instance, an excess of Fe or Cu can lead to increased production of toxic oxygen radicals via the Fenton and Haber-Weiss reactions [23]. Non-essential heavy metals (e.g., Cd, Hg, As) are generally toxic because they displace appropriate metals from enzymes or react with active thiol residues in proteins. Phytochelatin synthase is found in plants and a wide variety of other organisms, from cyanobacteria, algae, ferns, fungi, nematodes [18], [24]. Phytochelatins synthesized by PCS are involved in the chelation of a variety of heavy metals.

Little is known about the regulation of metal availability in schistosomes or the detoxification of toxic heavy metals [25]. Because schistosomes live in an iron-rich environment, regulation of iron is the best characterized of the metals [26]. Previous studies have found that iron is stored in female worms in yolk ferritin [27], [28] and iron acquisition in schistosomes has been shown to be accomplished by divalent metal transporters [29].

Here we present an initial characterization of the *S*. *mansoni* (Sm)PCS gene and protein. We found that three *S. mansoni* PCS transcripts are produced by alternative splicing from the unique SmPCS gene, potentially encoding three different PCS proteins. Two of these proteins containing the complete phytochelatin synthase domain resulted in large enhancements of tolerance to cadmium toxicity when expressed in *Saccharomyces cerevisiae*. We also found that this tolerance required free GSH. Phytochelatins containing 2–5 repeat units are produced by recombinant SmPCS expressed in *S*. *cerevisiae*. Multiple SmPCS mRNAs are expressed in all the mammalian phases of *S*. *mansoni* life cycle. Expression of SmPCS increases when *ex vivo* worms are cultured in media containing cadmium, iron, copper, or zinc. Collectively, this study indicates that SmPCS plays an important function in the schistosome-host interaction and is a potential candidate for drug development against schistosomiasis.

## Materials and Methods

### Parasite Preparation

Infection of mice (NIH Swiss, National Cancer Institute) with *S*. *mansoni* cercariae (NMRI strain) obtained from infected *Biomphalaria glabrata* snails and perfusion of adult worms (6–7 wk) from mice were done as described [30]. This study was approved by the Institutional Animal Care and Use Committee at Rush University Medical Center (IACUC number 08–058; DHHS animal welfare assurance number A3120-01). Rush University Medical Center's Comparative Research Center (CRC) is operated in accordance with the Animal Welfare Act {(Public Law (P.L.) 89–544) as amended by P.L.91–579 (1970); P.L.94–279 (1976); P.L. 99–198 (1985); and P.L 101–624 (1990)}, the Public Health Service's Policy on Humane Care and Use of Laboratory Animals (revised,2002), the Guide for the Care and Use of Laboratory Animals (revised, 1996) and the U.S. Government Principles for the Utilization and Care of Vertebrate Animals Used in Testing, Research and Training. The CRC is registered with the Animal and Plant Health Inspection Service (APHIS) arm of the United States Department of Agriculture (USDA). The Institution has an Animal Welfare Assurance on file with the National Institutes of Health, Office of Laboratory Animal Welfare (OLAW), A-3120-01. The facilities are accredited by the Association for Assessment and Accreditation of Laboratory Animal Care International (AAALAC International). The CRC is directed by the Senior Director of the CRC, a Doctor of Veterinary Medicine (D.V.M.) and a Diplomate of the American College of Laboratory Animal Medicine (ACLAM), who reports to the Associate Provost and Vice President for Research, who is also the Institutional Official for Animal Care and Use.

### RNA isolation and cDNA synthesis

RNA was isolated from adult worms collected from mice using the TRI reagent (Sigma-Aldrich), subsequent chloroform extraction and isopropanol precipitation of RNA following the manufacturer's instructions. The quality and quantity of the RNA was checked by A_260_/A_280_ in a Schimadzu UV-1800 spectrophotometer. Complementary DNA (cDNA) was synthesized using 1 µg RNA, 1 µl oligo dT (500 µg/ml), 1 µl 10 mM dNTP mix along with the 0.1 M DTT, 1 µl RNaseOut and 1 µl reverse transcriptase (200 unit) in the Superscript II Reverse Transcriptase Kit (Invitrogen) following the manufacturer's protocol.

### Cloning of SmPCS transcripts and analysis of alternate splicing

An adult worm cDNA library (kindly provided by Dr. Philip LoVerde) was used as a PCR template to amplify the SmPCS open reading frame using Taq DNA polymerase and gene-specific primers (See Table 1 for primer sequences). PCR product was cloned into pCRII TOPO TA vector (Invitrogen). Modified 5′RACE was performed using the T3 primer of pBlueScript II or the trans-spliced leader primer [31] and a reverse, gene-specific primer. PCR products ligated in pCRII vector were transformed into TOP 10 *Escherichia coli* strain following the manufacturer's protocol (Invitrogen) and plated on LB agar plates overnight at 37 °C with 50 µg/ml kanamycin. Plasmids were isolated using Qiagene mini plasmid isolation kit and sequenced by Applied Biosystems 48 Capillary 3730 XL DSL Analyzer. Sequencing reactions were done using 100 ng of template plasmid DNA, the M13 forward or reverse primer and Big Dye Terminator V3.1 Cycle Sequencing at DNA service facility of University of Illinois at Chicago (www.uic.edu/depts/rrc/dnas/).

**Table 1:** PCR primers used in this study.

**Primers used for cloning SmPCS in pYES 2.1 vectors**	
Mitochondrial SmPCS	**F** 5′-GTAATGGGCAATACTACACTATCACTTTCCG-3′
	**R** 5′-TCACTTTTGCTCTACACAACCTGTAC-3′
N-truncated SmPCS	**F** 5′-GTAATGGGCTCTGCAGTATGTGATGC-3′
	**R** 5′-TCACTTTTGCTCTACACAACCTGTAC-3′
PCS gene with partial PCS domain	**F** 5′-GATATGGGAATTCTAAATGCCTTAGG-3′
	**R** 5′-TCACTTTTGCTCTACACAACCTGTAC-3′
C-truncated SmPCS gene	**F** 5′-GTAATGGGCAATACTACACTATCACTTTCCG-3′
	**R** 5′-TCAATCAGTGGGTTTAGTCCAAGAATCTG-3′
**Primers used to study alternating splicing of SmPCS gene**	
Forward Primer from: i) Vector (T3)	**F** 5′-AATTAACCCTCACTAAAGGG 3′
ii)Non-codingN-terminus of SmPCS gene	**F** 5′-CTGACATCCAAACTGCTCAGCTCTAC-3′
Reverse Primers from coding region of SmPCS gene	**R** 5′**-**GTTGTTACGGTTTAGATGTGGA-3′
	**R** 5′-ATCCTGATACACAACTACCCCGA-3′
**Primers used in real time quantitative PCR**	
SmPCS	**F** 5′-GATGCCCCCAAGTTTCAGACT-3′
	**R** 5′- TGGTCGGCGGTAGAACTCTT -3′
GAPDH	**F** 5′-TGGTCGTATCGGGAGACTTGT-3′
	**R** 5′-CGAAACTACATCCACGGTGTTC-3′
**Primers used for reverse transcriptase PCR from different developmental phases of worm**	
Mitochondrial SmPCS	**F** 5′-CCAAATCAAAGTATGTCTGCAGTATGTGATG-3′
	**R** 5′-TCGGGGTAGTTGTGTATCAGGAT-3′
Cytoplasmic SmPCS-1	**F** 5′-GCAATCCAAATCAAAACTGATGTCATGCCA**-**3′
	**R** 5′-CAAGTTCTCTATCAGGGTGATAAC-3′
Cytoplasmic SmPCS-2	**F** 5′- TTACTCATAATAAAGGAAGAACCTTCTTAT-3′
	**R** 5′-GAAGTTCCTTTTGAGCTTTAACC-3′
GAPDH	**F** 5′-CAAGATGTCGAGAGCAAAGGTTGG-3′
	**R** 5′-CATGATGCGTTAGAAACCACGG-3′

### Expression analysis of SmPCS by reverse transcriptase PCR

The expression patterns of SmPCS transcripts were analyzed in different stages of the worm life cycle. Total RNA was isolated from eggs, schistosomula, juvenile liver worms, and male and female adult worms using the TRI reagent (Sigma-Aldrich) and cDNA was prepared as described above. One µl of cDNA was used as a template for PCR amplification. Forward primers were designed such that each set of primers would amplify only one transcript of the differentially spliced gene (Table 1). For the mitochondrial transcript, the forward primer was designed from a 35 nucleotide region of exon 2 that is spliced out of the two cytoplasmic transcripts (SmPCS-1 and SmPCS-2). To amplify SmPCS-1 only, the forward primer was designed having half of the primer sequence coming before the 35 nucleotide spliced region and the other half from the sequence after the 35 nucleotide spliced out region. For the SmPCS-2 transcript, the forward primer was made by taking the N-terminal half of the sequence from exon 1 and the other half from exon 3. The reversed primers were designed downstream of the forward primer sequences with a comparable primer melting temperature to amplify 690, 553, and 566 base pairs products from mitochondrial transcript, cytoplasmic transcript 1 and 2 respectively. Glyceraldehyde-3-phosphate dehydrogenase (GAPDH, GenBank accession M92359) was also amplified as the PCR template loading control.

### Expression of SmPCS during the mammalian life cycle of *S*. *mansoni* determined by quantitative PCR

Total RNA was isolated from the different stages of *S. mansoni* with TRI reagent (Sigma-Aldrich) and then cDNA was made as described above. One µl of cDNA was used as a template in triplicate assays for quantitative (q)PCR using the SYBR Green PCR Core Reagents and ABI PRISM 7900 sequence detection system (Applied Biosystems) following manufacturer's instructions. Primers were also designed for *S. mansoni* GAPDH to use as the internal control. For graphical representation of qPCR data, raw cycle threshold (ΔCt values) obtained from schistosomula, liver stage, female and male worm transcripts were deducted from the ΔCt value obtained for egg worm transcripts using the delta-delta Ct (ΔΔCt) method [32], [33], with GAPDH transcript levels serving as the internal standard.

### Analysis of the expression of SmPCS in adult *S*. *mansoni* worms in the presence of heavy metals

Adult worms collected by perfusion from mice were incubated in RPMI 1640 medium (Gibco) containing 25 mM HEPES (pH 7), 100 µg/ml streptomycin and 100 U/ml penicillin for 4 days. Cadmium (II) chloride, iron (III) chloride, zinc (II) chloride or copper (II) sulfate at 50 or 100 µM were added to the culture medium. Six pairs of worms per well were incubated in 6-well tissue culture plates at 37 °C with 5% CO_2_. Media and salts were replaced with fresh solutions after 48 hours. After 4 days worms were collected for RNA extraction, followed by the qPCR as described above.

### Expression of recombinant *S*. *mansoni* PCS proteins in yeast and cadmium tolerance assay

*Saccharomyces cerevisiae* strain K601 was selected to study cadmium tolerance in presence and absence of variants of recombinant SmPCS. The strain is uracil deficient. The ORFs encoding SmPCS mitochondrial or cytosolic variants were cloned into the yeast expression plasmid pYES 2.1 (pYES2.1 TOPO TA Expression kit; Invitrogen), which has the GAL promoter site. PCR amplification from adult worm cDNA was done using 1 unit *Pfu* DNA polymerase (Stratagene) following manufacturer's instructions. To add the A-overhang for cloning the PCR products in pYES2.1, the PCR products were treated with 2.5 unit of Taq DNA polymerase (Promega) for 12 minutes at 72°C. The primers used for cloning are listed in Table 1. The sequences of recombinant plasmids were verified. Plasmids were transformed into yeast cells following the instructions in cloning kit. Control yeast cells were prepared carrying empty pYES2.1 vector. Yeast strains were cultured in uracil deficient SC minimal medium following manufacturer's instructions. Recombinant protein was expressed by addition of 2% galactose as the carbohydrate source. For the cadmium tolerance assay, yeast strains were cultured in SC medium deficient of uracil containing CdCl_2_ from 0 to 1000 µM at 30 °C for up to 72 hours. Cell growth was monitored by A_600_. The DL-buthionine-(S,R)-sulfoximine (BSO, Sigma Aldrich) solution was prepared by dissolving the BSO in autoclaved water and sterilizing by filtration through a 0.44 micron filter (Millipore).

### Determination of PC production: HPLC analysis

Yeast strains expressing SmPCS proteins were grown for 48 hours at 30 °C in 500 ml SC minimal medium with 2% galactose. The cells were collected by centrifugation and then broken in 1.5 ml of Cellytic Y (Sigma-Aldrich). The supernatants were cleared by centrifugation and 75 µl were injected for HPLC analysis after dilution by an equal amount of water. HPLC analysis was done on a Jupiter C18 column (Torrance) using an HP1100 HPLC system (Agilent Technologies) at the University of Illinois-Chicago proteomics core facility. Mobile phase A consisted of 0.1% trifluoroacetic acid in Milli-Q water and mobile phase B consisted 0.1% trifluoroacetic acid in acetonitrile. The column was equilibrated with 5% solvent B. After sample injection, the column was eluted with a linear gradient from 5% solvent B to 100% solvent B in 40 min. at a flow rate of 1 ml/min. Elutes were collected and assayed with 500 µM 5,5′-dithiobis-(2-nitrobenzoic acid) (DTNB) in 50 mM potassium phosphate (pH 8) buffer to detect the free sulfhydryls of cysteine, γ-GluCys, GSH and PCs at 412 nm in Multiskan Spectrum plate reader [34], [35], [36]. Synthetic PC2 (γ-GluCys-γ-GluCysGly), PC3 (γ-GluCys-γ-GluCys-γ-GluCysGly) and PC4 (γ-GluCys-γ-GluCys-γ-GluCys-γ-GluCysGly were prepared at the proteomics core facility at the University of Illinois-Chicago.

### Determination of PC production: mass spectrophotometric analysis

The HPLC elutes that were positive in the DTNB assay were selected for mass spectrometric analysis. Cyano-4-hydroxycinnamic acid (CHCA) was used as the matrix for matrix-assisted laser desorption/ionization time of flight mass spectrometric analysis (MALDI-TOFMS) of the peptide solutions. Sample solutions were mixed 1∶1 with the matrix solution (10 mg CHCA in 1 mL aqueous solution of 50% acetonitrile containing 0.1% TFA). Aliquots (2 µl) were spotted onto a MALDI-TOF target and analyzed using a Voyager-DE PRO mass spectrometer (Applied Biosystems) equipped with a 337 nm pulsed nitrogen laser. Peptide mass was measured using positive-ion linear mode over the range m/z 50–3000. Analyses were conducted at the University of Illinois-Chicago proteomics core facility

## Results

### Genomic Analysis of SmPCS

**Figure 1:**
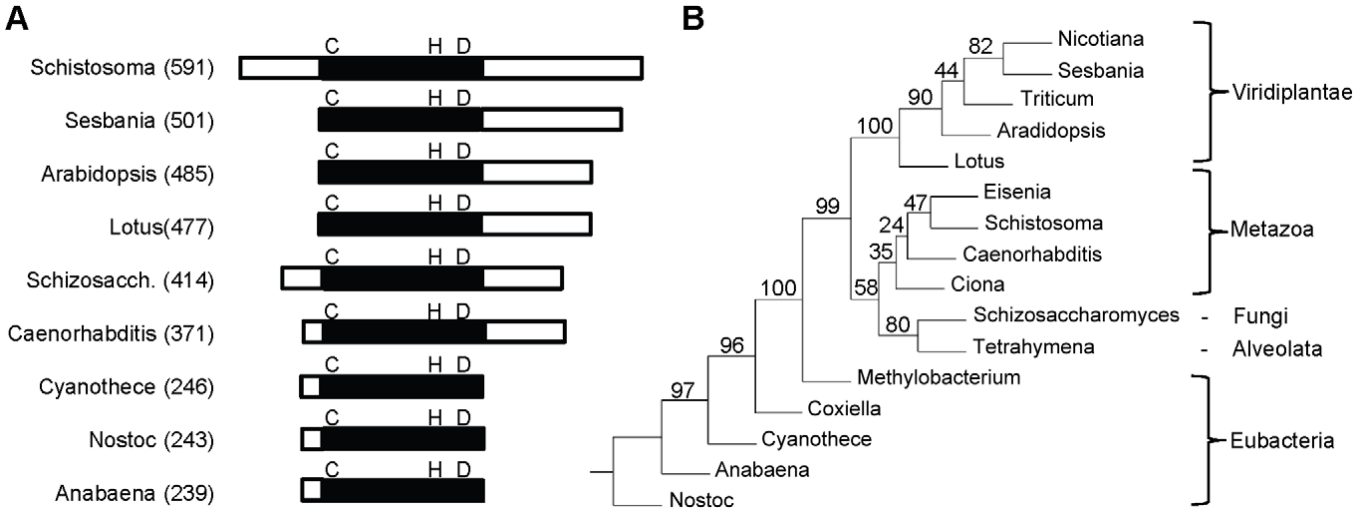
Genomic analysis of *Schistosoma mansoni* PCS. (A) Diagram showing difference in length between the PCS proteins from different organisms: The extended N terminal regions and the C- terminal extensions are shown as open boxes. The conserved PCS domains are shown as a filled boxes and the catalytic triad of cysteine-histidine-aspartic acid is indicated in single letter code. The total number of amino acids for each protein is indicated in parentheses. (B) A phylogenic tree based on amino acid sequence of the phytochelatin domains of selected PCS proteins. The genera and accession numbers for the PCS proteins used in the analysis are indicated. The unrooted, neighbor-joining tree was constructed using PHYLIP (http://bioweb.pasteur.fr/phylogeny/intro-en.html) [63] and bootstrap values (100 resamplings) are shown. The PCS protein sequences are *Nicotiana tabacum* (AAO74500), *Sesbania rostrata* (AAY82881), *Triticum aestivum* (AAD50592), *Arabidopsis thaliana* (AAF428050), *Lotus japonicus* (AAT80341), *Eisenia fetida* (ABR13683), *Schistosoma mansoni* (XP_002569764), *Caenorhabditis elegans* (AAK62991), *Ciona intestinalis* (XP_002128372), *Schizosaccharomyces pombe* (NP_593552), *Tetrahymena thermophila* AAY68362), *Methylobacterium nodulans* (YP_002490343), *Coxiella burnetii* (ABS78257), *Cyanothece* sp. (EDX97627), *Anabaena variabilis* (ABA22569), and *Nostoc punctiforme* (ACC81212).

Analysis of the *S*. *mansoni* genome sequence [37] indicated that there is a single PCS protein encoded in the genome (Smp_072740; *Schistosoma mansoni* Genome Project http://www.genedb.org/Homepage/Smansoni). One genome scaffold (Smp_scaff000249) encodes the entire PCS gene. No orthologous or parologous sequences were identified in the *S*. *mansoni* genome databases. The SmPCS gene encodes a predicted protein of 591 amino acids with a theoretical pI of 7.81 and Mw of 67,355 Daltons, which is considerably larger than PCS from other eukaryotes (Fig. 1A). The larger size of SmPCS is due to both N- and C-terminal extensions. Alignment of the PCS domain of SmPCS (the N-terminal half of the protein) with PCS domains from other organisms indicates that there is high sequence identity (36%–53%) and conservation of the catalytic triad of Cys, His and Asp in SmPCS (Figure S1). The identity of the PCS domain of SmPCS with the bacteria PCS proteins is lower, only 15%–26%. In addition, three of four Cys residues in the N-terminal portion of eukaryotic PCS proteins thought to bind cadmium and in some way be involved in the activity or activation of PCS [38] are present in SmPCS. An unrooted neighbor-joining tree (Fig. 1B) shows that SmPCS is phylogenetically most related to PCS from other metazoans, with PCS from plants, bacteria, protozoa and yeast clustering on separate branches.

### Alternative splicing of SmPCS transcripts

**Figure 2:**
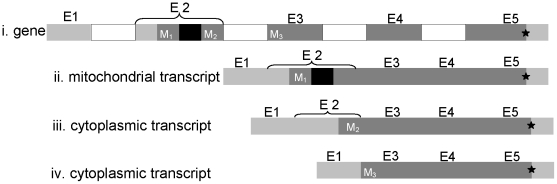
Alternative splicing of *Schistosoma mansoni* PCS transcripts. Three different mRNAs are formed by alternative splicing of the SmPCS primary transcript. i) The *S*. *mansoni* PCS gene. Exons 1 to 5 are shown as grey boxes, with the coding regions of exons in dark grey and the non coding parts in light grey color, and introns are shown as white boxes. Exon-1 is the 5′ non-coding region for all *S*. *mansoni* PCS transcripts. Potential initiator methionines are indicated as M_1_, M_2_, and M3. The stop codon, shown as a star (★), is same for all transcripts and is located in exon 5. ii) A transcript encoding a potential mitochondrial PCS protein. The start methionine, M_1_, is in exon 2. iii) A transcript encoding a potential cytoplasmic PCS protein. Thirty-five nucleotides are spliced out (black box) from exon-2 causing M_1_ and the mitochondrial targeting peptide to be present in a different reading frame from the reminder of the protein. The likely initiator methionine for this transcript is shown as M_2_ in exon-2. iv) A splice variant that results in the loss of the cysteine in the catalytic triad. In this transcript exon 2 is completely spliced out and exon 1 is directly spliced to exon 3. The predicted initiator methionine, M_3_, is present in exon 3 downstream of the catalytic cysteine residue.

Analysis of the SmPCS gene using a variety of predictive bioinformatic tools suggests that the gene product may be targeted to the mitochondria. MitoProt II 1.0a4 predicts SmPCS has a probability of export to mitochondria of 0.9063, the Predotar v. 1.03 value for mitochondrial targeting is 0.19, about 2x above expected value, and TargetP 1.1 predicts SmPCS has a probability of export to mitochondria of 0.857. There is also strong, but not conclusive prediction that mitochondrial SmPCS is secreted through a non-classical secretory process (Secretome 2.0, NN-score = 0.491; non-classically secreted proteins should obtain an NN-score exceeding the normal threshold of 0.5). Using adult worm cDNA as the template, three alternatively spliced SmPCS transcripts were identified (Fig. 2). The complete SmPCS gene has 5 exons and 4 introns. Exon 1 was present in all transcripts as part of the 5′ non-coding region of the gene. The transcript that encodes the complete SmPCS ORF (591 amino acids) has a 12 amino acid long mitochondrial leader sequence, including the start methionine residue, in exon 2. One cytosolic SmPCS variant (SmPCS-1) is formed by alternative splicing resulting in the deletion of 35 nucleotides from exon 2. This alternative splicing results in the mitochondrial targeting sequence being present in a different reading frame than the predicted protein with translation starting at a different methionine in exon 2. Complete splicing out of the second exon generated the second cytosolic transcript of SmPCS (SmPCS-2), which lacks the Cys residue of C-H-D catalytic triad. The predicted start methionine for this splice variant is located in exon 3. Using the trans-spliced leader sequence in reverse transcription-PCR there is no evidence that SmPCS transcripts are trans-spliced (results not shown).

### Analyses of the expression of PCS in *S*. *mansoni*

**Figure 3:**
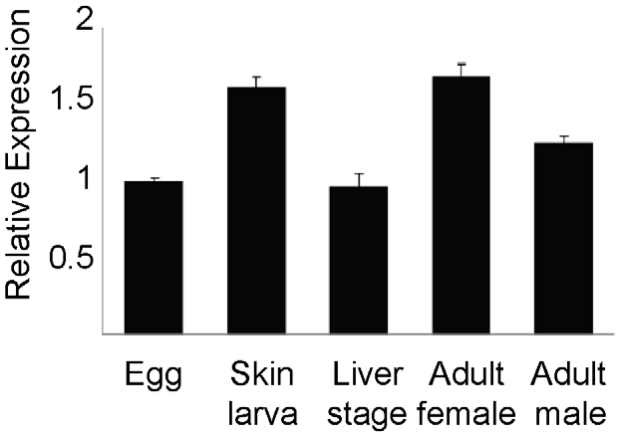
*Schistosoma mansoni* PCS transcripts in different stages of worm life cycle. Quantitative reverse transcription (q)PCR amplification of PCS transcripts was performed in triplicate on two biological replicates. For graphical representation of qPCR data, raw cycle threshold (Ct values) obtained for the different stages were deducted from the Ct value obtained for egg transcript levels using the deltadeltaCt (ΔΔCt) method [32], [33], with glyceraldehyde phosphate dehydrogenase (GAPDH) expression levels serving as the internal standard. Values are normalized as fold-difference relative to the egg stage. RNA was isolated from adult female worms, F; adult male worms, M; schistosomula, Sc; eggs, E; and liver stage juvenile worms, L.

Expression of the SmPCS gene in different stages of the worm life cycle was determined using quantitative reverse transcription-PCR (qPCR). GAPDH was used as the internal control. qPCR was done in triplicate on two biological replicates and is represented graphically (Fig. 3). Expression was found to be slightly higher in adult worms and schistosomula than in eggs and liver-stage worms. Due to the high degree of similarity between the three transcripts it was not possible to make specific primers to run qPCR for each transcript individually. Similar difficulties were encountered in designing primers for qPCR to distinguish the four PCS transcripts of *Sesbania rostrata* [39]. The results of reverse transcription-PCR showed that all three transcripts are present with approximately the same abundance in the five stages of the life cycle analyzed (results not shown). We confirmed the specificity of the primers used in the reverse transcriptase PCR by running separate PCR reactions using the sequenced clones in plasmid as template, specific for a particular transcript. The transcript specific primers were only capable of amplifying the expected sized products when transcript specific clones were used as the template (data not shown).

### Expression of SmPCS in adult *S*. *mansoni* in response to heavy metals

**Figure 4:**
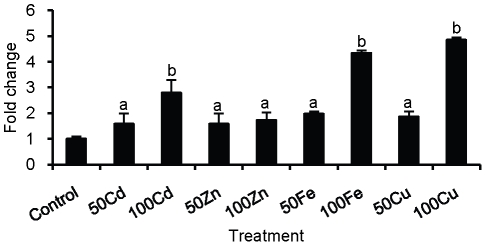
PCS transcripts expression in adult *ex vivo Schistosoma mansoni* in response to heavy metal exposure. Quantitative reverse transcription (q)PCR amplification of PCS transcripts was performed in triplicate on three biological replicates. For graphical representation of qPCR data, raw cycle threshold (Ct values) obtained for the different stages were deducted from the Ct value obtained for to transcript levels in control worms not exposed to any metals using the deltadeltaCt (ΔΔCt) method [32], [33], with glyceraldehyde phosphate dehydrogenase (GAPDH) expression levels serving as the internal standard. The concentrations of the heavy metal salts tested were 50 µM (50) and 100 µM (100). Bars labeled ‘a’ are significantly higher (p<0.05) than control; bars labeled ‘b’ are significantly higher (p<0.05) than the control and ‘a’.

Adult *ex vivo* worms were cultured in the presence of heavy metals and SmPCS expression was analyzed by qPCR using the ΔΔCt method with GAPDH as the internal control (Fig. 4). In the presence of heavy metals an increase in PCS expression compared to non-treated control was found. The highest increase in expression, almost 5 fold, was seen when worms were cultured with 100 µM iron or copper. Almost a 3-fold increase in expression occurred in worms incubated with 100 µM cadmium. At 50 µM concentration of metals, the expression of SmPCS was significantly above the control, between 1.5–2 fold. A smaller, but significant increase in PCS expression was seen in response to zinc exposure at both 50 µM and 100 µM of metal exposure.

### Tolerance to cadmium toxicity conferred by expression of SmPCS in yeast

**Figure 5:**
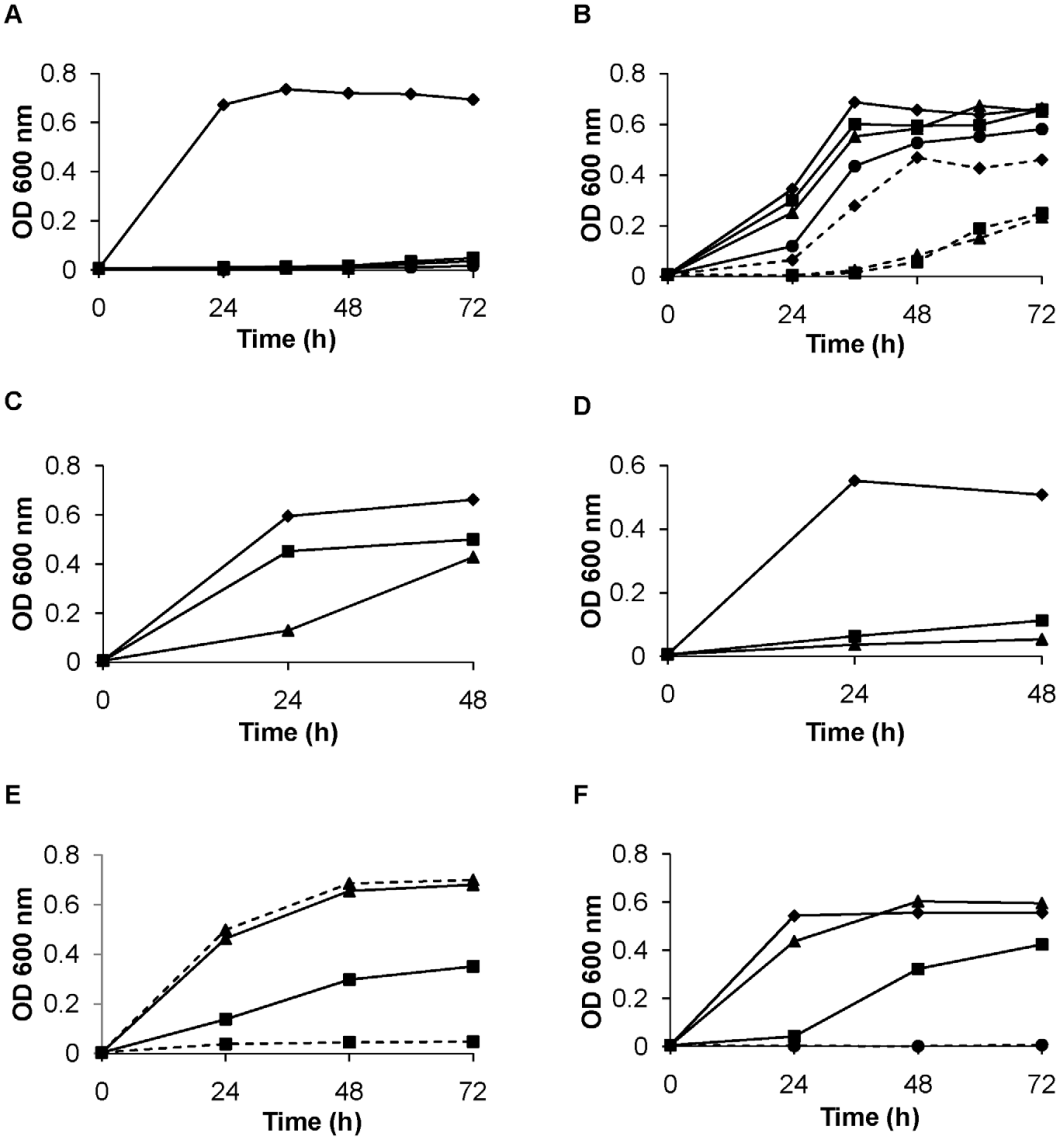
Functional characterization of *Schistosoma mansoni* PCS. The growth of yeast, monitored by the increase in turbidity, transformed with the (A) empty vector (pYES) or the (B) mitochondrial variant of *S*. *mansoni* PCS in the presence of different concentrations (0–1000 µM) of cadmium chloride (0 µM, —♦—; 50 µM, —▪—; 100 µM, —▴—; 250 µM, —•—; 500 µM, ––♦––; 750 µM ––▪––; 1000 µM ––▴––). The growth of yeast transformed with the N-truncated variant of *S*. *mansoni* PCS (C) or the C-truncated variant of *S*. *mansoni* PCS (D) in 0 µM (♦), 250 µM (▪), or 500 µM (▴). (E) The growth of yeast transformed with the mitochondrial variant of *S*. *mansoni* PCS (PCS, solid lines) or the PCS variant with a partial PCS active site lacking the catalytic cysteine residue (PCS-P, dashed lines) in 0 µM (▴) or 500 µM (▪) cadmium chloride. (F) The growth of yeast transformed with the mitochondrial variant of *S*. *mansoni* PCS with no additions (♦), in the presence of 500 µM L-buthionine sulfoximine (BSO, ▴), an inhibitor of γ-glutamyl cysteine synthase, the first enzyme in the glutathione synthesis pathway, 500 µM cadmium chloride (▪), or both 500 µM BSO and 500 µM cadmium chloride (•). Yeast were cultured in SC minimal medium minus uracil plus 2% galactose at 30°C. The cell growth was monitored by spectroscopy at 600 nm.

*Saccharomyces cerevisiae* was chosen to investigate the activity of SmPCS because it is sensitive to cadmium toxicity and produces negligible amounts of phytochelatins [16], [40], [41]. When the mitochondrial variant of SmPCS gene is expressed in yeast, cadmium tolerance is dramatically increased compared to yeast cells carrying the empty vector (Fig. 5A). Yeast cells expressing mitochondrial SmPCS were capable of growth in 1000 µM CdCl_2_ (Fig. 5B). By comparison, control cells carrying the empty vector were unable to grow in 50 µM CdCl_2_ (Fig. 5A). To investigate the importance of N-terminal and C-terminal ends of SmPCS, N-truncated SmPCS was made by deleting the first 65 N-terminal amino acids of the SmPCS, including the mitochondrial signal sequence. When assayed in yeast, the growth of cells expressing the N-truncated SmPCS protein (Fig. 5C) was the same as yeast cells expressing the mitochondrial SmPCS (Fig. 5B). A C-truncated SmPCS construct was made by deleting the C-terminal 100 amino acids. Yeast carrying C-truncated SmPCS gene were unable to grow in the presence of CdCl_2_ above 50 µM (Fig. 5D). It should be noted that both the N- and C-truncated proteins contain the complete phytochelatin synthase activity domain. The SmPCS cytosolic transcript that lacks the Cys residue of C-H-D catalytic triad was also assayed in yeast for the cadmium tolerance. Under the same growth conditions, yeast carrying this transcript was highly sensitive to cadmium exposure (Fig. 5E). These results suggest that SmPCS with the complete catalytic triad is capable synthesizing PC, which then scavenges and neutralizes cadmium permitting yeast growth. If phytochelatins are formed, there should be a dependence on GSH in the cadmium tolerance induced by SmPCS expression. To examine if the GSH is involved, 500 µM L-buthionine sulfoximine (BSO), an inhibitor of γ-glutamyl cysteine ligase, the first step in GSH synthesis [12], was added to yeast cell cultures carrying the mitochondrial variant of the SmPCS gene. BSO or CdCl_2_ alone caused no reduction of yeast growth. However, cell growth was greatly reduced when both 500 µM BSO and 500 µM CdCl_2_ were present in the culture medium (Fig. 5F). The same result was found for the yeast cells expressing the cytoplasmic variant of the SmPCS protein having the complete PCS domain (data not shown). These results suggest that SmPCS synthesizes phytochelatins from GSH when expressed in yeast.

### Detection and identification of the phytochelatins formed in yeast by SmPCS activity

**Figure 6:**
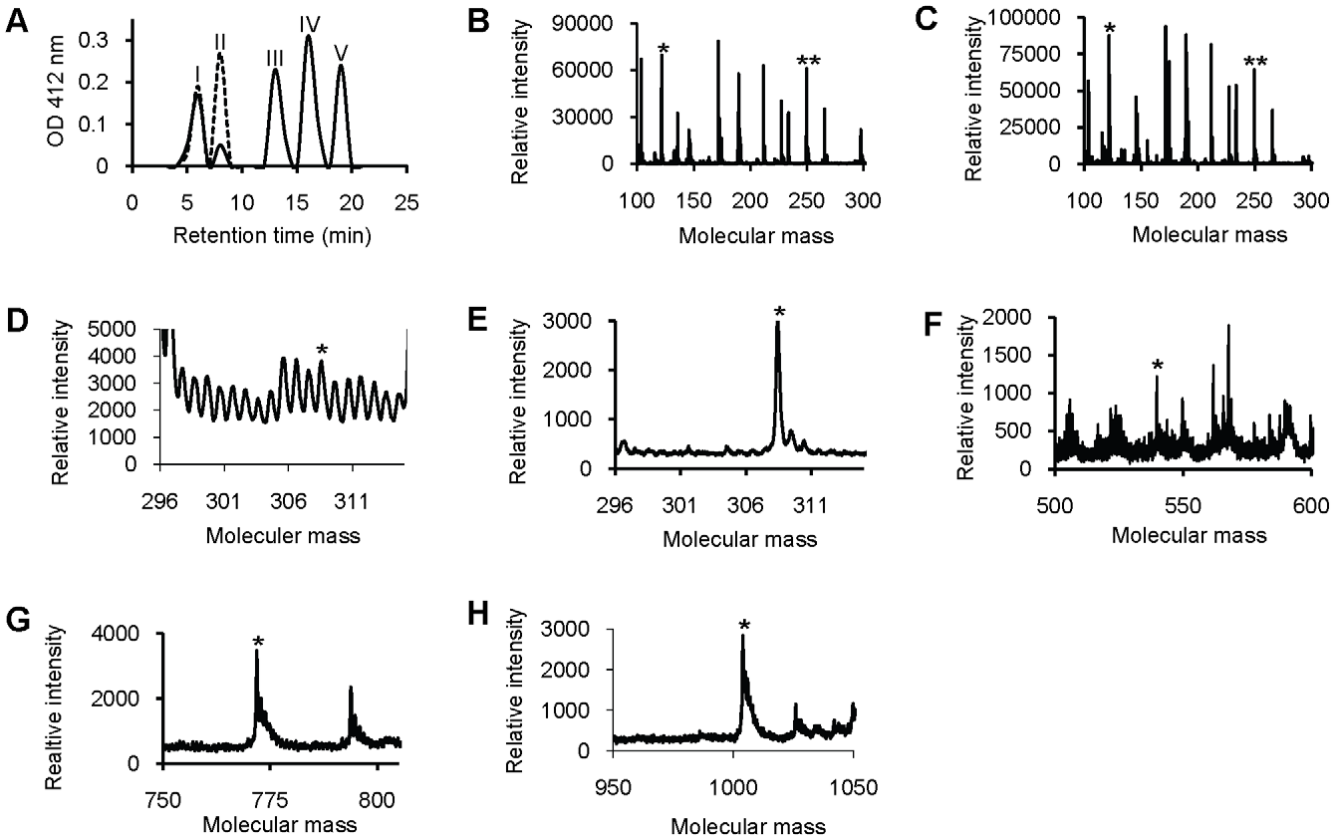
Detection of phytochelatins in yeast extracts by HPLC-MS. (A) Analysis of yeast cell extracts by HPLC and detection of reduced thiols using 5,5′-dithiobis-(2-nitrobenzoic acid) (DTNB). Extracts from yeast transformed with the mitochondrial variant of *S*. *mansoni* PCS (PCS, solid lines) or the empty vector (pYES, dashed lines) were fractioned by HPLC as described in Materials and Methods. Thiols in the fractions react with DTNB to release thionitrobenzoic acid, which is monitored by its absorption at 412 nm. Yeast cells were grown in SC minimal medium minus uracil plus 2% galactose at 30°C for 72 hours. Fractions indicated in (A) containing reactive thiols (peaks I–V) were analyzed by mass spectroscopy as described in Materials and Methods. Spectra from peak I from both PCS (B) and pYES (C) cultures indicate that cysteine (*) at 121.72 Da and γ-glutamyl-cysteine (**) at 249.6 Da are present. Spectra from peak II from both PCS (D) and pYES (E) cultures indicate that glutathione is present at 308.48 Da (*). Spectra from peaks III (F), IV (G), and V (H) found only in extracts from PCS cultures indicate that phytochelatins with two repeats (γ-EC)_2_G (*, 539.76 Da), three repeats (γ-EC)_3_G, (*, 771.87 Da) and four repeats (γ-EC)_4_G (*, 1003.98 Da) are present, respectively.

Yeast strains carrying full-length SmPCS gene or empty vectors were grown for 48 hours at 30°C in presence or absence of 500 µM CdCl_2_ in yeast induction medium. Cell extracts were fractionated by HPLC and the HPLC elutes were analyzed by the classical DTNB assay [34] (Fig. 6). The results clearly show that five peaks (fractions 5, 7, 12, 15 and 18) were generated from yeast cell extracts expressing SmPCS gene while the yeast cell extracts made from cells with the empty vector had two peaks (fractions 5 and 7). Synthetic PCs fractionated by HPLC under the same conditions were identified in the same fractions as shown for the yeast extracts in Figure 6 (results not shown).

DTNB-reactive fractions were selected for further examination by mass spectroscopy for peptide identification. MS analysis identified the presence of Cys and γ-GluCys in fractions 5 and GSH in fractions 7 of both yeast cell extracts (Fig. 5). It appears that phytochelatin production in yeast cell expressing SmPCS protein significantly depleted GSH levels as a smaller GSH peak is seen compared to this sample from yeast cells with empty vector. Thiol-reactive peaks in fractions 12, 15 and 18 were only found in yeast cells expressing SmPCS. Mass spectrometric analysis detected phytochelatins with 2 (γ-GluCys-γ-GluCysGly), 3 (γ-GluCys-γ-GluCys-γ-GluCysGly) and 4 (γ-GluCys-γ-GluCys-γ-GluCys-γ-GluCysGly) repeats of γ-GluCys in fractions 12, 15, and 18, respectively (Fig. 6). Identification of phytochelatins by mass spectrometry confirms the activity of recombinant SmPCS in the production of PCs that can then act as cadmium scavengers. Synthetic PCs were found at the same MW by MALDI-ToF as shown for the yeast extracts in Figure 6 (results not shown).

## Discussion

We have shown that a homologue of PCS is present in *S*. *mansoni*. Genome analyses indicate that humans and other mammals do not have genes for PCS. In mammals, heavy metal availability/sequestration is accomplished by metallothionines and GSH [42]. It has recently been determined that humans use GSH, not PCs, for an ATP binding cassette (ABC transporter) to detoxify cadmium [43]. Because PCS is absent from the human genome, we hypothesized that schistosome PCS may be a suitable drug target for schistosomiasis treatment. To establish this, we present here an initial characterization of *S*. *mansoni* PCS. We find that SmPCS has conserved amino acid residues in its active site suggesting that the catalytic mechanism of SmPCS should be similar to characterized PCS proteins. When expressed in yeast, SmPCS provides protection from cadmium toxicity, allowing yeast to multiply in the presence of high concentrations of cadmium in a GSH-dependent manner. SmPCS is expressed in all mammalian lifecycles stages and its expression is increased in response to the presence of heavy metals. Collectively, these results indicate that PCS plays an important role in the regulation of heavy metal availability in *S*. *mansoni*.

Although the overall similarity of SmPCS to other PCS proteins is relatively low, on the order of 28%, if one considers only the most conserved, N-terminal half of the proteins, identity is ∼45%, which is similar to the conservation seen when comparing plant, fungal and nematode PCS proteins. When the complete PCS domain is present in the SmPCS construct expressed in yeast, with or without the mitochondrial targeting sequence, large increases in cadmium tolerance were observed, with growth occurring even in 1000 µM cadmium. However, expression of a form of SmPCS with truncation of the 100 C-terminal amino acids (but with the complete PCS catalytic site) does not provide cadmium tolerance. The C-terminal domains of PCS proteins from eukaryotic organisms are poorly conserved and their function in enzyme activity has not been clearly defined. It has been speculated that the C-terminal region of PCS proteins may have functionally diverged in individual organisms. PCS in cyanobacteria, which lack the C-terminal domain, have been reported to primarily catalyze the hydrolysis of GSH and GSH conjugates, in which GSH is converted to γ-glutamylcysteine and γ-glutamylcysteine S-conjugates [20], [21]. However, the C-terminal domain in PCS from eukaryotes has a role in PCS synthesis since an *Arabidopsis thaliana* PCS mutant truncated in the C-terminal domain has a cadmium-sensitive phenotype and C-terminally truncated PCS has decreased thermal stability and responsiveness to heavy metals [44]; [45]. It has been suggested that heavy-metal binding by cysteine residues present in the C-terminal domain have a role as a sensor for heavy metals [46]; [47]; [48]. These cysteine residues may function by binding the heavy metal with its subsequent transfer into the closer proximity with the catalytic domain [49]. The C-terminal domain in SmPCS contains eight cysteines and deletion of the one hundred C-terminal amino acids removed five of these cysteines. Expression of this mutant protein did not allow the growth of yeast in cadmium suggesting that this region plays an important function in synthesis of PCs by SmPCS. Alternatively, deletion of this region of the protein resulted in a PCS protein with poor stability and a short half-life. The precise functions of the C-terminal region of SmPCS remain to be determined.

PCS proteins are structurally and catalytically related to Clan CA cysteine proteases including papain and lysosomal cathepsins from animals [15]. Both classes of enzymes have a requirement for a nucleophilic cysteine, which is made more nucleophilic by a 3D-proximal histidine residue [15]. The third residue in the catalytic triad of cysteine proteases is an asparagine, which is sometimes replaced by an aspartic acid [50]. In PCS proteins, an aspartic acid aligns with the conserved asparagine/aspartic acid in cysteine proteases. Site-directed mutagenesis was used to determine that the catalytic triad of Cys-56, His-162 and Asp-180 was absolutely required for phytochelatin synthesis in *A*. *thaliana* PCS [51]. Recently, the structure of a prokaryotic PCS-like protein was described confirming that PCS proteins belong to the papain superfamily of cysteine proteases and display conservation of the 3D geometry of the catalytic cysteine-histidine-aspartic triad [14]. The catalytic cysteine-histidine-aspartic acid triad is conserved in SmPCS. Deletion of the cysteine of the triad results in a protein that is unable to confer resistance to cadmium toxicity in yeast, indicating that a similar catalytic mechanism must occur in SmPCS as in other PCS proteins. However, transcripts spliced so that they lack this catalytic cysteine were identified in cDNA populations from adult *S*. *mansoni* worms. The function of proteins expressed without a complete catalytic triad is not clear. It should be noted that alternative transcript splicing resulting in partial deletions of the catalytic triad has been seen in other organisms as well. The tropical legume *Sesbania rostrata* has been reported to have four transcripts including two that lack the complete catalytic triad. These variants were not able to confer cadmium tolerance when expressed in *S*. *cerevisiae* [39]. Splice variants of PCS genes in *Lotus japonicus* have also been reported [52]. We find that all three PCS transcripts, including cytoplasmic transcript-2 that lacks that the complete catalytic triad, are expressed in all the life stages of *S*. *mansoni* interacting to its human host. Expression of the cytoplasmic transcript 2 is intriguing but its function remains to be determined.

Although it is difficult to imagine that *S*. *mansoni* parasites are routinely exposed to elevated levels of toxic heavy metals within the controlled environment of their definitive host, dietary influx could potentially expose adult and juvenile liver worms in the hepatic portal system to elevated levels of both essential and non-essential heavy metals. In addition, schistosomes degrade host hemoglobin as a source of both heme and amino acids. However, excess heme is toxic due to the ability of its reduced iron to generate oxygen radicals and other toxic reactive species. The polymerization of heme to a nontoxic, insoluble waste product, hemozoin, is important for schistosomes and other hematophagic parasites [53]. SmPCS may be crucial for heavy metal sequestration in the worm. We hypothesize that PCS and phytochelatins are involved in the detoxification of iron produced during the breakdown of host hemoglobin in the parasite gut. Several metals, notably Cu, Fe, Mn, and Zn, are cofactors in essential metalloenzymes within the mitochondria [54]. Since some PCS appears to be targeted to mitochondria, it may have a role metal homeostasis in this cellular compartment in schistosomes.

Reverse genetic approaches have been used to verify the role of PCs and PCS in resistance to heavy metals in a number of systems. RNA interference silencing of PCS in *C*. *elegans* produced a cadmium hypersensitive phenotype [16]. Deletion of the *pcs* gene in *Schizosaccharomyces pombe* produced strains that were ten-times more sensitive to cadmium and more sensitive than the wild type to arsenic, but no increased sensitivity to copper, zinc, mercury, selenium, silver, or nickel was seen [55], although others found increased sensitivity to copper in *S*. *pombe pcs* knock outs [41]. Arabidopsis *cad1* mutants are cadmium-hypersensitive and deficient in phytochelatins [55] and are mutated in the gene for PCS1 [55]. Attempts to silence SmPCS in adult *S*. *mansoni* worms have not been successful thus far (results not shown). However, the role of SmPCS in defense against heavy metals may be inferred from analysis of factors inducing its expression. Therefore, it was of interest to know if SmPCS expression shows any changes after exposure of worms to heavy metals. Worms exposed to cadmium had increased SmPCS expression. Increased PCS expression also occurred in response to exposure to copper and iron and to a lesser extent to zinc. These results indicate that SmPCS is involved in the processes to regulate the availability of copper and iron, potentially including iron released from heme, and suggests a role in the detoxification of other heavy metals. Increases in PCS expression by copper, iron and zinc has been reported in a variety of plants [35], [56]–[58].

Our investigations suggest that the SmPCS protein has similar activity to PCS proteins from other organisms. Expression of *S*. *mansoni* PCS in all life stages of *S. mansoni* interacting with its human host strongly suggests that it is an essential gene and therefore it can be considered as a prospective target for new drugs. Previous studies have found significant increases in iron storage proteins and zinc transporters in skin stage parasites relative to cercariae [59] reinforcing the importance of metal homeostasis in schistosomes. The drugability of PCS proteins is unknown. No PCS inhibitors are known because their identification has not been the priority of previous research. The structural relationship of PCS proteins to cysteine proteases could be exploited to identify and develop inhibitors of PCS. Cysteine proteases are currently targets for many diseases including cancer, inflammatory diseases, malaria, Chagas disease, schistosomiasis and other parasitic diseases [60]–[62] and chemical libraries targeting cysteine proteases are available. We could potentially tap into the rich array of known cysteine-protease inhibitors to identify PCS inhibitors. This is a goal of our future studies on *S*. *mansoni* PCS. Since both parasitic nematodes and trematodes (and potentially cestodes) have PCS genes/proteins, the identification of compounds targeting PCS activity could have broad impacts on drug development for a number of important human pathogens, which are largely neglected by the pharmaceutical industry.

## Supporting Information

Figure S1 **Multiple sequence alignment of the phytochelatin synthase domain containing the active site of** ***S*** **.** ***mansoni*** **PCS with the phytochelatin synthase domains from other organisms.** The amino acid residues of phytochelatin domains were aligned using ClustalW multiple sequence alignment program. Identical residues are shown with a black background and conservative changes are shown with gray background. The conserved catalytic triad of in the phytochelatin domains, C-H-D, are shown in red and cysteine residues thought to be involved in cadmium binding are shown in yellow. One cysteine residue is substituted by a lysine in *S*. *mansoni* PCS and is indicated by a blue circle. The accession numbers for the sequences used are shown in Figure 1B.
(TIF)
