# Supplementary figures and images for: Characterization of the Phytochelatin Synthase of Schistosoma mansoni
Source: PLoS Negl Trop Dis. 2011 May 24;5(5):e1168. doi: 10.1371/journal.pntd.0001168 (PMC3101182; doi:10.1371/journal.pntd.0001168)

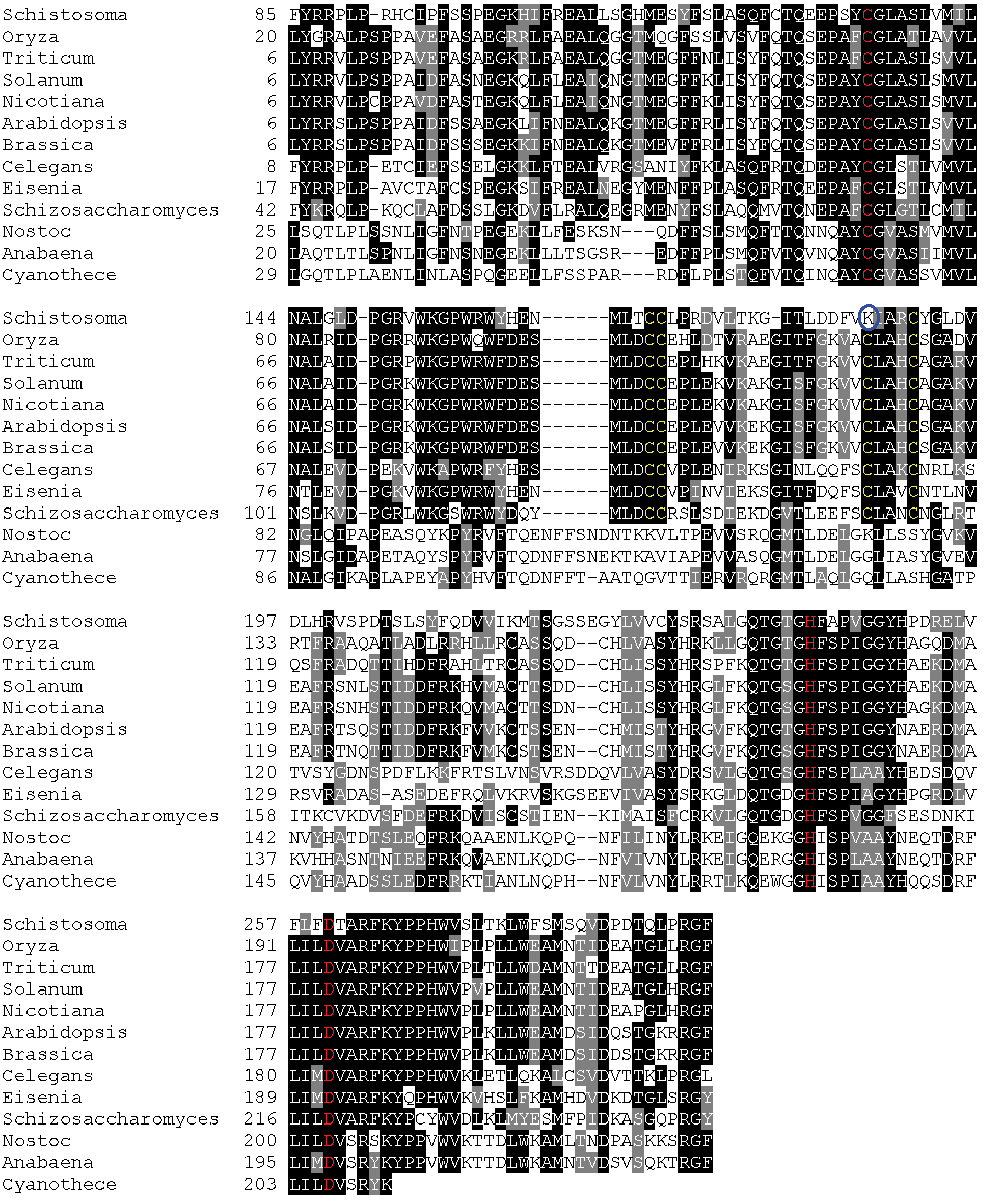

Supplement: Figure S1 — Multiple sequence alignment of the phytochelatin synthase domain containing the active site of S . mansoni PCS with the phytochelatin synthase domains from other organisms. The amino acid residues of phytochelatin domains were aligned using ClustalW multiple sequence alignment program. Identical residues are shown with a black background and conservative changes are shown with gray background. The conserved catalytic triad of in the phytochelatin domains, C-H-D, are shown in red and cysteine residues thought to be involved in cadmium binding are shown in yellow. One cysteine residue is substituted by a lysine in S. mansoni PCS and is indicated by a blue circle. The accession numbers for the sequences used are shown in Figure 1B. (TIF) [file pntd.0001168.s001.tif]
